# An Efficient Defocus Blur Segmentation Scheme Based on Hybrid LTP and PCNN

**DOI:** 10.3390/s22072724

**Published:** 2022-04-01

**Authors:** Sadia Basar, Abdul Waheed, Mushtaq Ali, Saleem Zahid, Mahdi Zareei, Rajesh Roshan Biswal

**Affiliations:** 1 Department of Information Technology, Hazara University Mansehra, Mansehra 21120, Pakistan; sadiaa.khancs@gmail.com; 2 Department of Computer Science, Abbottabad University of Science and Technology, Abbottabad 22010, Pakistan; 3 Department of Computer Science, Northern University, Nowshera 24100, Pakistan; abdul@netlab.snu.ac.kr; 4 School of Electrical and Computer Engineering, Seoul National University, Seoul 08826, Korea; 5 Institute of Computer Science & Information Technology, The University of Agriculture, Peshawar 25130, Pakistan; szahid77@gmail.com; 6 School of Engineering and Sciences, Tecnologico de Monterrey, Zapopan 45201, Mexico; m.zareei@tec.mx

**Keywords:** defocus blur, EDAS, in-focused region, LTP, out-of-focused region, PCNN

## Abstract

The defocus or motion effect in images is one of the main reasons for the blurry regions in digital images. It can affect the image artifacts up to some extent. However, there is a need for automatic defocus segmentation to separate blurred and sharp regions to extract the information about defocus-blur objects in some specific areas, for example, scene enhancement and object detection or recognition in defocus-blur images. The existence of defocus-blur segmentation algorithms is less prominent in noise and also costly for designing metric maps of local clarity. In this research, the authors propose a novel and robust defocus-blur segmentation scheme consisting of a Local Ternary Pattern (LTP) measured alongside Pulse Coupled Neural Network (PCNN) technique. The proposed scheme segments the blur region from blurred fragments in the image scene to resolve the limitations mentioned above of the existing defocus segmentation methods. It is noticed that the extracted fusion of upper and lower patterns of proposed sharpness-measure yields more noticeable results in terms of regions and edges compared to referenced algorithms. Besides, the suggested parameters in the proposed descriptor can be flexible to modify for performing numerous settings. To test the proposed scheme’s effectiveness, it is experimentally compared with eight referenced techniques along with a defocus-blur dataset of 1000 semi blurred images of numerous categories. The model adopted various evaluation metrics comprised of Precision, recall, and F1-Score, which improved the efficiency and accuracy of the proposed scheme. Moreover, the proposed scheme used some other flavors of evaluation parameters, e.g., Accuracy, Matthews Correlation-Coefficient (MCC), Dice-Similarity-Coefficient (DSC), and Specificity for ensuring provable evaluation results. Furthermore, the fuzzy-logic-based ranking approach of Evaluation Based on Distance from Average Solution (EDAS) module is also observed in the promising integrity analysis of the defocus blur segmentation and also in minimizing the time complexity.

## 1. Introduction

**Figure 1:**
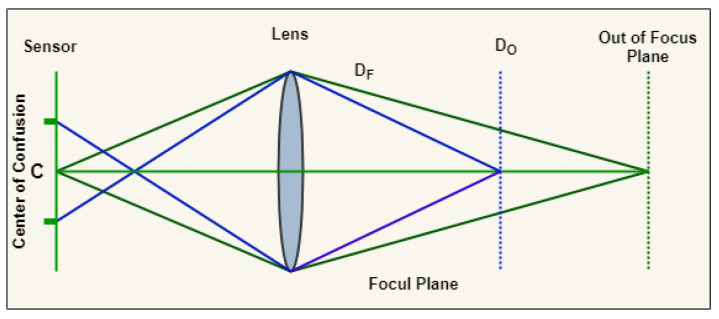
Circle of Confusion in focal and out of focus plane.

Defocus blur using the optical-imaging system is the output of the focused region in an image. In the image creation system, the light radiation on the in-focus plane incoming from the object points is plotted to a focal point in the optical sensor. Nonetheless, the light coming from an object point outside an in-focus plane illuminates a non-object point of a region on an optical sensor is known as a Circle-of-Confusion (CoC) as illustrated in Figure 1. This concept is called defocus blur when the CoC is as large as noticed by our eyes. The main importance of defocus blur images compared to the regular image is that it consumes less space in the memory as it has color compressions in the background.

It is worth mentioning that there is no sharp boundary in defocus images that splits the image into sharp and blur regions. The defocus blur highly depends on the diameter of the camera lens, which recommends that narrow lenses generate lower defocus and tend to lower blurry backgrounds. Contrarily, wider camera-aperture lenses cause wider defocus. The in-focused region is usually the foreground area of the optical image, and it is sharp while the remaining part is considered background, the out-of-focused region that protects the viewers from distraction, and for this purpose, wider aperture lenses are used by photographers. Out-of-focused images compromise the image quality, and it is assumed a detrimental impact and misses the essential details necessary for the image scene interpretation. Consequently, robust and automatic in-focused and out-of-focused region detections are essential for numerous applications, including object detection, image scene classification, image segmentation, and defocus depth estimation. The in-focused or sharp region is commonly known as the Region of Interest (RoI) that automatically grabs the audience’s attention. These images are also used for retrieval and searching the object of interest in similar images. For the accurate and efficient segmentation of defocus blur, there is a need for adaptive feature extraction from the sharp region and segmentation algorithms without degrading the in-focused region [1].

The latest image defocuses algorithms presume that the blurry region is the output of spatially invariant [2,3,4,5,6,7]. Algorithms that standardize the spatially-invariant defocus commonly restore small local patches of the defocus images, where the blurry region is observed as the invariant, and also the restored patches of the image are mutually stitched [8,9,10]. A robust in-focused and out of focused region detection is worthwhile in numerous contexts comprising: (i) avoiding inaccurate post-processing of out of focus regions such as deconvolution [10]; (ii) identification of blurred background in digital photography containing depth map estimation, image de-blurring, quality analysis of the image, and further blur effect can also be adding for deploying artistic-bokeh effect [11,12], (iii) object recognition in microscopy images where the main object is blurred, and it needs to be detected using proper feature extraction such that microscopic images [13].

**Figure 2:**
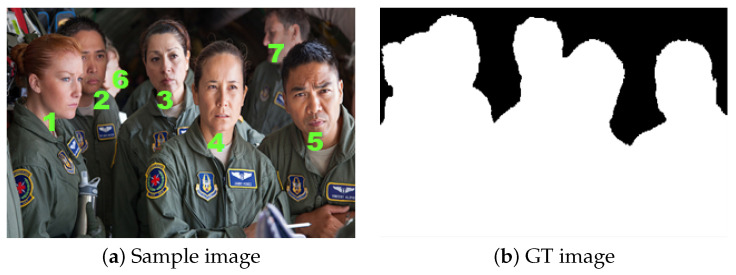
The figure represents the two images, i.e., the original sample image along with its respective GT image. The sample image numerically identifies the blurred and non-blurred objects: the non-blurred objects are represented by 1, 2, 3, 4, and 5, respectively, whereas the rest are blurred objects. In the GT image, white indicates the non-blurred objects, whereas the black spots identify the blurred region.

One of the foremost motivations of defocus-blur segmentation is to segment the blurred and non-blurred regions to facilitate post-processing, as noticed above. Our study explicitly adopted this research problem; a hybrid and novel scheme comprises Pulse Coupled Neural Network, and a sharpness map-based Local Ternary Patterns are proposed. The proposed scheme outperforms in terms of yielding smooth shapes of the object along with its visible edges, producing prominent and efficient focused results for the common datasets and also used in blurred and noisy images than previous alternatives [14,15,16,17,18,19,20,21,22,23]. Figure 2 illustrates the numerical representation of in-focused and out-of-focused patches in the sample image: the labeling of in-focused objects are 1, 2, 3, 4, and 5, respectively, while the remaining are denoted as out of the focused region. In the ground-truth (GT) image, black spots indicate the blurred region, while white indicates the in-focused region. Contributions of the current work include:

- We proposed a novel and simple yet effective hybrid scheme by adopting a sharpness-based descriptor known as Local Ternary Patterns (LTP) and a pulse synchronization technique called Pulse Coupled Neural Network (PCNN) for addressing the defocus blur segmentation limitations. Moreover, the threshold-based positive values are the prerequisite for the region extraction process in defocus blur segmentation.
- The LTP-based sharpness metric is applied instead of the LBP descriptor (prone to noisy background and blur regions) for accurate extraction of partially blur regions by estimating the blur area in defocus blur images.
- Next, the Pulse Coupled Neural Network algorithm uses the neuron-firing sequence phenomena after the region extraction that consists of the pixel feature details, i.e., edges, texture, and region, which are used for a noticeable out-of-focused region segmentation using the de-blur image features.
- The experimental results are evidence that the proposed blur-measure produces promising results while using limited computation time and processing in various de-blur environments.
- To estimate the ranking of the referenced and proposed schemes using the appraisal scores $\left({ℜ}_{a}\right)$ calculated for the defocus blur segmentation using fuzzy logic-based EDAS approach for different performance metrics including precision and recall, and F1 measure.

We adopted the fuzzy approach in this study to classify proposed and referenced algorithms in their proper ranking category. The image segmentation-based fuzzy methods are majorly involved in numerous areas, for example, fuzzy set theory, genetic algorithm, supplier segmentation [24], pattern recognition, neural networks, computer vision, and image processing [25]. Ghorabaee et al. [26] proposed a technique based on Fuzzy Logic (FL), called Evaluation Based on Distance from Average Solution (EDAS). It is a scheme of Multiple Criteria-Decision-making Method (MCDM), used for the classification of inventory [27]. The FL-based EDAS approach is usually applied to rank numerous algorithms to appropriately classify the top algorithms concerning the accuracy, time, and speed. It is observed from Table 10 that the proposed scheme is ranked on top, while Zeng [23], and LTP [22] are positioned on the second and third rank, Basar21 [21], and Zhu [19] on the fourth and fifth rank. LBP [20], Shi14 [16], Su [17], Shi15 [14], and Zhuo [18] are ranked on sixth, seventh, eighth, ninth, and tenth, respectively.

### Paper Organization

The paper organization is classified as follows. Section 2 represents the related work of defocus-blur images and the PCNN method along with LBP, LTP, and EDAS approaches. Section 3 contains the proposed scheme followed by the proposed defocus-blur segmentation algorithm, its implementation steps, and algorithm description. The evaluation of the segmentation results obtained by the proposed scheme along with the dataset, comparator algorithms, EDAS ranking approach, and discussion is described in Section 4. Finally, Section 5 explains the conclusion.

## 2. Literature Review

In this section, first, the Depth-of-Field (DoF) images are briefly introduced and followed by the referenced work of the PCNN technique. Subsequently, comprehensive literature is provided about the LTP and LBP schemes and the EDAS-based approaches in various application fields.

### 2.1. Defocused Blur Image Segmentation and PCNN Technique

This section mentions the introduction about Low-Depth-of-Field (LDoF) images and the PCNN technique. The Depth-of-Field (DoF) is a metric measurement that depends on the camera distance between its anterior lens angle and also the posterior object angles, which is sharp [28]. In LDoF images, when the distance is low and the image features are sharp, then it means the Object of Interest (OoI) is deblurred. Conversely, the blurred region covers the background with a large volume, which in turn is a loss of detail in large areas [29]. The classical approaches for OoI detection in LDoF images are region-based, edge detection techniques, and features transformation. The edge detection algorithm explained in [30,31] detects the object’s artifacts and also performs the analysis of the object’s contouring to determine the defocused-blur pixels. On the contrary, the region-wise segmentation algorithm is required for object detection in images of natural scenes [29,32,33,34,35] by exploring a region along with high-frequency techniques. For instance, the latest works [36,37,38] apply a higher-order statistics (HOS) measure from out-of-focused scenes to explore the in-focused regions. A multiscale fuzzy model is implemented by Mei et al. [39] for the identification of RoI, while the mixed-energy function is used for defocus-blur region segmentation.

In the same way, a multiscale deblurring approach is implemented by Shaik et al. [40] for out-of-focused region detection in the saliency space for curve evolution method in LDoF images. Likewise, a multi-focused-fusion technique is proposed by Roy and Mukhopadhyay [41] for focused edges extraction from the input sample image to achieve a better fusion focused image. They applied zero-cross and Canny operators for edge detectors to obtain a prominent extraction of focused edges. Contrarily, the feature-transform segmentation of LDoF images yields better results, followed by an improvement in performance. Nonetheless, the extraction of the defocused object of a particular LDoF image is costly in terms of computation. Similarly, Wen et al. [42] introduced a Convolutional-Neural-Network (CNN) of 2 channels for clarity map extraction of fusion images. The clarity map is smoothed by adopting a morphological-filtering method. Lastly, the prominent segments of sample images are combined to construct the fused image as output.

However, motivated by the feature transformation segmentation techniques for defocused-blur images, our attention has been turned into PCNN and LTP-based algorithms for extracting the defocus-blur region of defocused-blur images. The PCNN is a lightweight algorithm because it does not require training like other neural-based network models. PCNN algorithm has some well-known image processing features, like, pulse-synchronization and global coupling [43].

PCNN adopted a paradigm based on bio-inspired parallel processing for handling problems of complex nature. Eckhorn et al. [44] presented the PCNN technique as a novel phenomenon in artificial neural networks research, whereas the source of its concept is the visual mammalian neurons for providing the pulses of synchronization. The PCNN is a single layer, and a 2D array of lattice-linked neurons followed by a 1-to-1 correspondence of neuron pixels in such a way that each neuron is interconnected along with a novel pixel and vice versa [45]. The novel PCNN execution is required to reproduce the phenomenon of pulse burst in the visual cortex nervous structure of *cats.* It is applied in numerous application areas of computer vision, i.e., image processing and segmentation, object and pattern recognition and extraction, image enhancement, and fusion [29]. Recently, the exceptional characteristics of the PCNN algorithm have been applied for developing novel models for image segmentation and studying the dynamic-synchronization potentials based on neuron behavior, consisting of its capturing activity, synchronizing based pulse release, automated signal, and non-linear components. One of the impressive contributions of the PCNN model might be represented in the study of image segmentation in natural scenes, in medical imaging, and some other types of images [46], and consequently, our interest turned into evaluating its strengths and shortcomings.

However, a more systematic study about the applicability of the Pulse Coupled Neural Network (PCNN) method in the area of image segmentation is required to assess its relevancy and set proper adaptable mathematical parameters for that model. In this scenario, Deng et al. [47] evaluated the mathematical properties of PCNN for the first time. Subsequently, in [48,49], the research about the evaluation of the time reduction parameter was performed. An approach for analyzing parameters’ setting of connection-coefficient was presented by Kuntimad et al. [50]. Chen [51] proposed an adaptive method for settings of parameters for neural model simplification. Yi-De et al. [52] and Min and Chai et al. [53] suggested the Fisher and mutual criteria for enhancing the PCNN performance in the image segmentation area. Helmy and El-Taweel [54], Xu et al. [55], and Hernandez and Gómez [56] introduced an evolution differential and a self-organizing neural network approach, correspondingly for adaptability improvement of PCNN in numerous applications of image segmentation.

From a context point of view in our study, Shen et al. [13] introduced the application of PCNN in refocusing images for region detection. The refocusing images use defocus-blur region detection in 3-D analysis and appropriately measure the distance and depth. PCNN’s pulse-sequence result consists of important information, i.e., textures, edges, and plane regions in the image. One of the major shortcomings of the suggested approach is that it is used for light field images only.

The pixels’ spatial properties are measured while adopting the PCNN method in image segmentation. For example, the gray-scale pixel values are dissimilar, i.e., edge’s discontinuities. The most common applications of the PCNN method are considered in the industry uses because the output of the neuron firing system detects the features of the digital image, i.e., texture, edges, and deblur region [6].

This research applies the best properties of the PCNN algorithm for transforming edges and region features into neural pulse sequence images and extracting the in-focused region of the defocused-blur images from the pulse sequence. 

In digital optics, the defocus image regions are majorly classified into sharp, blurred, and transitive regions. Likewise, digital imaging features can be mainly classified into three types, i.e., color, texture, and shape. The defocus segmentation performs the main role in separating the sharp and blurred regions in digital images. A hybrid scheme consisting of a novel descriptor of sharpness based on LTP (Local Ternary Patterns) and the PCNN model is introduced for determining this type of problem.

### 2.2. Local Patterns Based Segmentation and EDAS Approach

This section discusses the literature about LTP, LBP, and EDAS schemes in more comprehensively defocus blur segmentation. LBP is prone to noise, whereas LTP, the extended version of LBP, resolved the noise issue and produced more noticeable results.

#### 2.2.1. Local Binary Pattern (LBP)

Ojala et al. [57] presented a novel descriptor for texture known as Local Binary Pattern (LBP). LBP determines the variations in the pixel values amid the central points and also the neighboring ones to generate a binary pattern. Such binary descriptor yields a decimal value which is utilized for labeling a specific pixel. It is formally described that for a particular pixel $z_{c}$, the comparison about LBP measure is performed along-with the *p* neighboring pixels $\{{z_{p,r}\}}_{n=0}^{p-1},$ on a circular radius *r* on $z_{c}$ centroid.

Yi and Eramian [20] proposed an LBP measure for defocus-blur segmentation to segment the in-focused and out-of-focused regions in LDoF images. In the presented technique, the out-of-focused regions are inaccurate in the LBP sharpness compared to in-focused regions. A quality result can be yielded by fusing the suggested approach following image multiscale and matting inference. The major shortcoming of the author’s proposed work is that its accuracy is greatly affected by noise in blurry images.

LBP requires simple execution steps and low computational complexity, which is the main reason for its common application in image analysis and computer vision. It is also broadly used for the detailed analysis of texture; its widespread applications with other numerous classifications are remote sensing area, CBIR, visual inspection of distinct objects, analysis of biometric images (face, palm, and fingerprint detection and recognition), edge detection, analysis of objects in motion, and texture recognition [58,59,60]. Pietikäinen et al. [61] suggested the LBP function about a certain pixel is described in Equations (1) and (2) as follows:

(1) $${LBP}_{p,r}\left(x_{cn}\right)=\sum_{n=0}^{p-1}M\left(z_{p,r,n}-z_{cn}\right)2^{n}$$

(2) $$M\left(x\right)=\;\left\{\begin{array}{c} 1\;\;\;\;x\geq 0\\ 0\;\;\;\;x<0 \end{array}\right.$$

where $z_{c}$ represents the central level pixel value, $z_{p}\left(p=0,\cdots ,p-1\right)$ illustrates the value of neighborhood pixels on a Circle of Radius (CoR) by *r* and the neighborhood pixels are indicated by *p*. The first-order-hold interpolation can assess the gray scale neighboring pixels $z_{p}$ that avoids falling in the central pixels.

Shi14 et al. [16] proposed a blur feature-based algorithm. For adjusting the scale variance, numerous blur modules are illustrated and merged in a multiscale component. The authors yield the defocused-blur dataset and ground-truth images for their future domains. One of the primary limitations of the suggested framework is that it is sometimes highly suffered in the case of background without texture or motion-blurred foreground images, resultant in the out-of-focus pixels are characterized in such kinds of regions. Shi15 et al. [14] presented a new sparse-feature based technique for estimating the just noticeable blur in deblurred images. A robust correspondence between the extracted features following the out-of-focus strength is described by the proposed method. The suggested approach illustrates numerous applications of well-trained features, containing a refocusing image, image deblurring, and estimating depth in images.

Basar21 et al. [21] proposed a novel and hybrid approach based on PCNN and LBP for segmenting the defocus images in focused and out-of-focused regions. The author’s proposed technique is adaptively adjusting the model’s parameters according to the image. Their proposed LBP descriptor and PCNN are applied to obtain the in-focused resultant images with visible regions and edges. The presented algorithm outperforms the existing methods in terms of accuracy and efficiency.

The prime limitation of the techniques mentioned above is that it severely affects under noisy pixels, mixes up the image content with the noise while structuring the descriptor, and has inaccurate performance in the presence of slight distortion in the image content. Therefore, there is a need for further improvement in blur performance detection. As a result, the LTP metric has an indeterminate state, and the compatible bit value is defined, which is based on an alternate descriptor that might require the apparent consideration about detection of noise in the blurry region of defocusing the image.

#### 2.2.2. Local Ternary Pattern (LTP)

**Figure 3:**
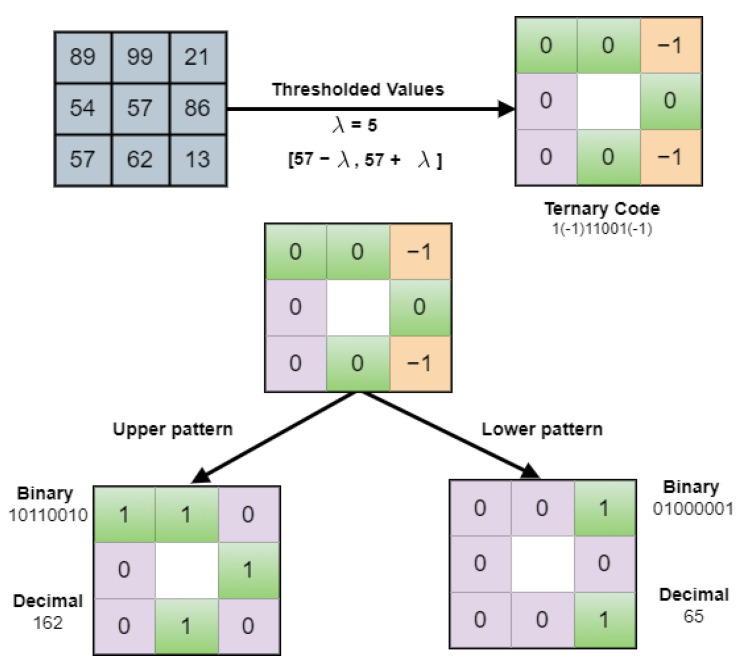
LTP descriptor.

Tan and Triggers [62] proposed an efficient texture operator approach that resolves the noise sensitivity issue involved in LBP. LTP is the extended version of LBP, which has a 3-valued code such as −1, 0, 1. One of the examples of the LTP descriptor is depicted in Figure 3. The LTP metric is described mathematically by [63] in Equation (3) and Equation (4) as given below:

(3) $${LTP}_{p,r}\left(y_{cn}\right)=\sum_{n=0}^{p-1}U\left(z_{p,r,n}-z_{cn}\right)3^{n}$$

(4) $$U\left(y\right)=\;\left\{\begin{array}{cc} 1 & y\geq \lambda \\ 0 & -\lambda <y<\lambda \\ -1, & y<-\lambda \end{array}\right.$$

where $z_{cn}$, and $z_{p,r,n}$ are explained in Equation (3) and λ indicates the subject threshold. After applying the thresholding step, the upper $\left({LTP}_{upp}\right)$ and lower patterns ${(LTP}_{low})$ are created and then coded. Khan et al. [63] stated that 0 in Equation (4) is the primary value for pixel allocation that lies in the range of threshold ±λ while 1 is the pixel allocation value if it is above the threshold λ and −1 if the value is below. The LTP descriptor is the combination of +LTP and −LTP values and is not viable in noisy images. So, the −LTP values are required either to be eliminated or to be changed into +LTP values for post-processing in the next step. For this purpose, the resultant *LTP* metrics are distributed upper ${(LTP}_{upp})$ and lower patterns ${(LTP}_{low})$. The values obtained in ${(LTP}_{upp})$ are binary bit-stream which need to be transformed into relevant decimal values as shown in Figure 3. The ${(LTP}_{upp})$ is obtained by transforming −1 into 0 and the rest have remained unchanged as depicted in Equation (5) below:

(5) $${LTP}_{upp}=\left\{\begin{array}{c} \;1\\ \;0\\ -1 \end{array}\to \left\{\begin{array}{c} 1\\ 0\\ 0 \end{array}\right.\right.$$

In the same way in $\left({LTP}_{low}\right)$, −1 is replaced into 1 and 1 into 0 as given in Equation (6).

(6) $${LTP}_{low}=\left\{\begin{array}{c} \;1\\ \;0\\ -1 \end{array}\to \left\{\begin{array}{c} 0\\ 0\\ 1 \end{array}\right.\right.$$

Tariq et al. [64] proposed LTP based blur segmentation for defocus-blur images. The presented algorithm performs the transformation of each pixel into a ternary code, which is later on converted into ${(LTP}_{upp})$ and ${(LTP}_{low})$. Similarly, the LTP along with moments are adopted by Srivastava et al. [22] for content-based image retrieval based on image features, i.e., color, texture, and shape. Conversely, Agarwal et al. [65] mentioned that single-modality features, for example, color, texture, and shape, are not sufficient for an accurate CBIR, so they suggested a method called multi-channel LTP based on novel features for a prominent CBIR. However, the performance of the above-presented frameworks for CBIR may degrade while using pure defocused-blur datasets.

Zeng et al. [23] adaptively estimated the blur map by applying the convolution neural networks (CNN) for testing and training of relevant local features of defocus-blur images using the local descriptor map. Anwar et al. [66] suggested features based on CNN of a patch-pooled set algorithm for estimating the depth and also removing blur from the single out-of-focus image. In this method, the focused image is restructured for blurriness removal and obtain synthetic refocusing from a single image. The author’s presented method is used for everyday images without getting any prior information. Zhao et al. [67] proposed an image-scale-symmetric cooperative network (IS2CNet) for defocus blur detection (DBD) in defocus images. The proposed model works twofold: firstly, IS2CNet expands the receipt of blur image content from the high to low image scale process. Therefore, the detection map of homogeneous regions is increasingly optimized. Secondly, from the high to low image scale process, IS2CNet observes the content of the over-exposed image, thus increasingly filtering the modification of region detection. They also proposed a hierarchical feature and bidirectional delivery technique that spread the feature of earlier image scale as an input and the track of present image scale for the network of present image scale guidance in the rest of the learning process. Zeng et al. [68] also introduced a method based on CNN known as deep residual convolutional encoder-decoder network (DRDN) for DBD. DRDN aims to adaptively produce pixel-based predictions in LDoF images and perform reconstructed detection results similar to the input sample image. Then various deconvolution operations through the transposed level convolution are performed at numerous image scales, and the further link is skipped. These approaches can efficiently perform blur region detection in defocus images. However, these algorithms are complex and detect some estimated outputs that contain inaccurately labeled regions compared to the ground truth images.

Su et al. [17] proposed an algorithm for blurry region detection and classification in partially blurred images that detected adaptively and classified the blur region types irrespective of applying kernel or deblurring estimation. A novel feature about the decomposition of singular value (DSV) is applied for image blurry region detection. The information about the alpha channel is used by the suggested method to categorize the blur type, for example, motion-blur or de-blur regions. The main limitation of the proposed approach is that the blurriness of the entire image might affect the estimated quality of blur degree as per the evaluation performed by DSV β1. One of the aspects of performance degradation is the ratio regarding the blurriness domain size to the full image size. Depth-map is another estimated model that may apply defocus blur segmentation. Zhuo and Sim [18] apply a novel scheme to estimate the blur-map at edge levels under the Gaussian ratio algorithm. The defocus map evaluation can be performed twofold: the first is to evaluate the number of blurry edges, and the second is to expand the blur estimation for the whole image using matting-interpolation to obtain the entire defocus-map. The quality of the depth-map estimation is highly dependent on the edge detection accuracy and blur map accuracy at the image edges.

Zhu et al. [19] used the point-spread function (PSF) for estimation to reveal the geometric mean of the image scene and also to improve the in-focused regions in the blurry image. The proposed technique used a certain defocus image for estimating a defocus map while working with standard cameras. The suggested approach produced the blur map by using edges’ color information to detect the blurred pixels.

Though the techniques above successfully detect the blur-map in defocus images, overall, the referenced algorithms face challenges in accurately segmentation of blurred and non-blurred smooth regions. The major limitations of the state-of-the-art blur detection techniques are inaccurate detection, complex algorithms, and prolonging the duration of blur detection. 

#### 2.2.3. EDAS Scheme

This study applied the Evaluation Based on Distance from Average Solution (EDAS) scheme to rank alternate schemes. The recent research works about the impacts of the EDAS based fuzzy logic scheme in different areas are specified in Table 1.

## 3. Method and Evaluation

The PCNN model contains pulse-coupled neurons, a 2-dimensional array model of monolayer neurons [13]. The neurons in the PCNN technique are identified as the pixels for their application areas in digital image segmentation. As the PCNN algorithm is based on the coupling nature of neurons, a neuron, i.e., pixel firing will follow the concurrent firing of those neurons belonging to the same class. The coupling nature of the PCNN model executes the images by applying numerous approaches.

**Figure 4:**
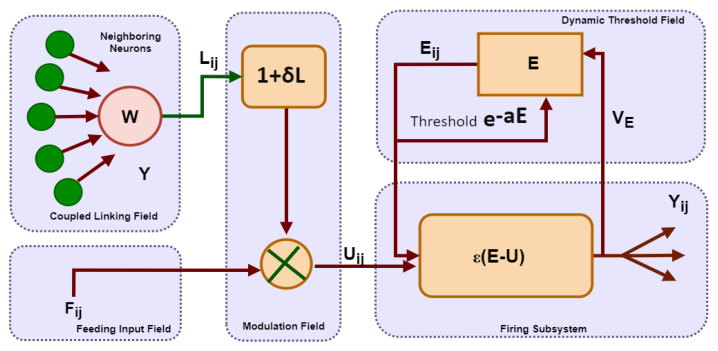
Schematic Structure of PCNN model.

The PCNN model is depicted in Figure 4. The mathematical representation of the five subsystems in the model is given as follows in Equations (7)–(11):

(7) $$F_{ij}\left(n\right)=Z_{ij}$$

(8) $${\;\;L}_{ij}\left(n\right)=Q_{L}\sum W_{ijkl}\Upsilon_{kl}\left(n-1\right)$$

(9) $${\;\;\;\;\;U}_{ij}\left(n\right)=F_{ij}\left(n\right)\left(1+\delta AL_{ij}\left(n\right)\right)$$

(10) $${\;\;\;\;\;\;E}_{ij}\left(n\right)=e^{-aE}E_{ij}\left(n-1\right)+V_{E}\Upsilon_{ij}\left(n-1\right)$$

(11) $$\Upsilon_{ij}\left(n\right)=\epsilon \left\{\begin{array}{cc} \left[E_{ij}\left(n\right)-U_{ij}\left(n\right)\right] & U_{ij}\left(n\right)\geq {Td}_{min}\\ 1 & U_{ij}\left(n\right)<{Td}_{min} \end{array}\right.$$

The coupled-linking subsystem is identified in Equation (7) whereas Equations (8) and (9) represent the input feeding sub-system. The dynamic thresholding sub-system and modulation are represented in Equation (10), while Equation (11) characterized the firing sub-system; $G_{ij}$ denotes the gray-level pixel linking to neuron; subscript ij identifies a particular pixel’s coordinates about a certain image, whereas the subscript kl signifies a neighboring pixel’s coordinates; δA denotes the internal activity’s modulation sub-system; dynamic-strength coefficient is represented by ϵ. The characterizations of $a_{E}$ and $V_{E}$ for the time constant about iterative-decay and amplification coefficient of linking-weight in the correspondence of dynamic threshold and the firing subsystem. The output state of the PCNN is represented by $\Upsilon_{ij}$ which is the neuron’s firing state that is 0 or 1. The in-focused region during the iterations in the model is represented by neurons firing naturally or triggered by neighboring neurons in image segmentation. This scheme is proposed for segmenting the in-focused region; each neuron is allowed for one-time firing in each iteration.

Meanwhile, the setting of minimum thresholding attenuation ${Td}_{min}$ ensured that all the neurons had the chance of getting fired. The setting of dynamic-thresholding coefficient ${ℑ}_{E}$ with the minimum limit means that all the remaining neurons will be fired. The estimation of gray-level values of adjacent pixels is directly proportional to the neighboring neurons firing. Likewise, the ordering of neurons firing indicates the gray-scale change in pixels’ values. The earlier knowledge of neuron firing was adopted in this research to estimate the regional properties of defocused-blur images. The PCNN neurons and their neighbors get fired with the help of the linking and feedback sub-system. The dynamic decay of the threshold of adjacent neurons is fired in the next step.

The Local Ternary Patterns (LTP) blur metric is broadly applied for face recognition [62], texture classification [75], and content-based image retrieval (CBIR) [22].

Suppose the blurry sample image is identified by $D_{Img}\left(z\right)$ whereas z=(x,y) represents the coordinates of a pixel in the domain of digital image ω. The blur pixel degree at location *x* is measured by using a blurred based operator $B_{o}$ in a neighboring local window across that particular pixel as given in Equation (12) as follows:

(12) $$l\left(z\right)=B_{o}\left(D_{Img}\left(z\right)\right)$$

The proposed blur metric is based on the LTP distribution in the in-focused and out-of-focused regions. For a central pixel $z_{cn}$, three values are assigned by LTP to the neighborhood pixels based on their central pixel intensity difference. Those intensities that come in the range of $\pm t_{d}$ are required to be assigned as zero, +1 is assigned to those that above of $n_{cn}+t_{d}$ while $n_{cn}-t_{d}$ is assigned by −1, whereas $n_{cn}$ is allocated as the intensity of $z_{cn}$. The LTP code about pixel $z_{cn}$ is explained in Equations (13) and (14) as below:

(13) $${LTP}_{i,r}\left(x_{cn},y_{cn}\right)=\sum_{n=0}^{I-1}M\left(z_{i,r}-z_{cn}\right){\times 3}^{n}$$

(14) $$M\left(x\right)=\;\left\{\begin{array}{cc} 1 & \left|y\right|\geq {Td}_{LTP}\\ 0 & \left|y\right|<{Td}_{LTP}\\ -1, & \left|y\right|\leq {Td}_{LTP} \end{array}\right.$$

where $z_{i,r}$ represents *i* neighboring pixels intensities positioned on *r* circle radius at central pixels $x_{cn},y_{cn}$, and a minor built-in threshold with a positive value ${Td}_{LTP}>0$ is used to achieve the region robustness for a flat digital image. It is not necessary for an intensity $z_{i,r}$ to be located at the central location in the pixels of the image, hence it is achieved with the help of bilinear interpolation. The local ternary pattern is decomposed into two halves, one is called upper LTP (${LTP}_{upp}$) whereas another is known as lower LTP (${LTP}_{low}$) in Equations (15)–(18) as defined below:

(15) $${LTP}_{i,r}^{upp}\left(x_{cn},y_{cn}\right)=\sum_{n=0}^{I-1}M\left(z_{i,r}-z_{cn}\right){\times 2}^{n}$$

where

(16) $$M_{upp}\left(x\right)=\left\{\begin{array}{c} 1,\;\;\;\;\;\;\;\;\left|y\right|>{Td}_{LTP}\\ 0,\;\;\;\;\;\;\;\;\;\left|y\right|<{Td}_{LTP} \end{array}\right.$$

and correspondingly,

(17) $${LTP}_{i,r}^{low}\left(x_{cn},y_{cn}\right)=\sum_{n=0}^{I-1}M\left(z_{i,r}-z_{cn}\right){\times 2}^{n}$$

where

(18) $$M_{low}\left(x\right)=\left\{\begin{array}{c} 1,\;\;\;\;\;\;\;\;\;\left|y\right|<{Td}_{LTP}\\ 0,\;\;\;\;\;\;\;\;\;\left|y\right|>{Td}_{LTP} \end{array}\right.$$

In these local descriptors, uniform-rotation-invariant is the pattern where a circular bits’ sequence does not allow more than two transitions, 0 to 1 and 1 to 0. The rest of the patterns are grouped like a single pattern, called a non-uniform-pattern (NUP). The sharpness descriptor of LTP has one of the main advantages: it is effective for monotonic-illumination transformation, which commonly arises in natural scene images. The symbol definitions of this research are reported in Table 2.

### 3.1. Proposed Algorithm

In this section, the proposed steps along with the proposed segmenting algorithm are elaborated and shown in Figure 6. First, the pre-processing step filters the image and then transforms it into a gray scale. The LTP mask to produce sharpness is described in the next step, whereas the out-of-focused region estimation is explained in the third step. The fourth step is expanded about the PCNN model structure, while the proposed algorithmic definition following Algorithm 1 is illustrated at the start of the section.

#### 3.1.1. Algorithm Description

The defocus-blur segmentation algorithm adopting the proposed scheme is described in Algorithm 1. Our presented scheme accepts the LDoF input image while yielding the prominent regions of the resultant image. Our algorithm comprises five main components; initialization of parameters, production of the firing sequence matrix, classification of pixels, LTP metric estimation, and segmentation quality evaluation of the prominent regions.

The algorithm proposed involves various parameters, i.e., connecting-weight matrix $W_{con}$, connecting coefficient of strength δ, dynamic-threshold coefficient ${ℑ}_{E}$, decay-factor $F_{E}$, threshold value along with minimum value $Td_{min}$, and parameter for criteria of judgment C. We calculate the preliminary value of $W_{con}$ through the experiment. Some other parameters such that δ, ${ℑ}_{E}$, δ, $Td_{min}$, and C are configured adaptively corresponding to the distribution of the gray-scale pixel in de-blur dataset. The connecting-weight matrix $W_{con}$ indicates the gray-scale intensity information and is transferred by the neighboring neurons of the central one. The impact of gray-scale pixels reduces as the central pixel distance is expanded. To initialize the matrix, the synaptic weights are denoted in the matrix following the constant values which are reported in Equation (19) as given below:

(19) $$W_{con(ij)}\left[\begin{array}{ccc} 0.5 & 1 & 0.5\\ 1 & 0 & 1\\ 0.5 & 1 & 0.5 \end{array}\right]$$

The interval of the neuron’s activation to firing in the PCNN approach is step-wise adopted. Tsai [30] describes that $F_{E}$ modifies the measuring matrix height of each firing stage, while ${ℑ}_{E}$ measures the numbering and width of each firing stage. For example, if $F_{E}$ reduces, then the firing narrowing step of the neuron decreases, its statistical property of coupling, and depicted the network behavior of the pulse delivery. Conversely, the smaller $F_{E}$ means, the greater the duration of algorithm iteration, which in turn highly decreases the efficiency of the algorithm. The neuron is consistent with the value of the pixel and the greatest gray-level value $D_{Img}$*max* in a complete image required to be fired for the starting iteration period. Hence, ${ℑ}_{E}$ is generally allocated as $D_{Img}$*max*. Furthermore, each neuron is allowed to be fired once to avoid the overlapping cycles of firing neurons. As the neuron fired, the thresholded value is adjusted to be infinity. Subsequently, the neuron is not allowed for firing in the algorithmic cycle, as reported in Equation (20). The sample defocus blur input image $D_{Img}$ is as normalized as the matrix *γ*. This procedure is known as the refractory firing period (pulsing in this situation) of the neuron.

(20) $${ℑ}_{E}\leftarrow \gamma$$

The proposed scheme adopts simplified pre-processing phases of the de-blur images that estimate the spatial frequency statistics, gray-scale statistical distribution, and gray-level normalization. We settled the parameters as per the obtained pre-processing output for further improvement of the adaptability nature of the proposed scheme. The parameter ${Td}_{min}$ is denoted as the gray-scale distribution in the whole input image for avoiding the ineffectual cycles. Thus, the pixel’s number alongside gray-level values in the interval of parameter [${Td}_{min},\;1\geq 94\%$] of the threshold’s pixels *γ* generated in an entire blurry image. The three characteristic regions, low-level, mid-level, and high-level frequency data extraction, perform the out-of-focus image. Each characteristic region contains an image block along with the greatest level of pixels in the output frequency band. The gray-scale mean values for low level is indicated as $M_{LF}$ medium level is $M_{MF}$, and high-level frequencies are denoted as $M_{HF}$ following the symbolized standard deviations $\sigma_{LF}$, $\sigma_{MF}$, and $\sigma_{HF}$, respectively. The algorithm applied for the defocus dataset required to fulfill the subsequent measures by using such kind of parameters: neurons with $M_{LF}$ fire at the consistent time; the continued fire is performed by neurons with $M_{MF}$, while a higher difference is reported in the neurons firing of $M_{HF}$.

The possible two criteria for the $M_{LF}$ region is that at first, the neuron firing at the iteration of ${nf}_{1}th$, corresponding to the pixel value of gray-scale $M_{LF}+\sigma_{LF}$. Secondly, the neuron firing at the iteration of ${nf}_{2}th$, corresponding to the pixel value of gray-scale $M_{LF}-\sigma_{LF}$. $F_{E}$ fulfills the below inequality criteria, as illustrated in Equations (21)–(23):

(21) $$M_{LF}+\;\sigma_{LF}\geq {ℑ}_{E}e^{-\left({nf}_{1}-1\right)F_{E}}$$

(22) $$M_{LF}-\;\sigma_{LF}\geq {ℑ}_{E}e^{-\left({nf}_{2}-1\right)F_{E}}$$

(23) $${nf}_{2}-{nf}_{1}\leq 1$$

To solve Equations (21)–(23), the output expression is given below in Equation (24):

(24) $$F_{E}=\ln\frac{M_{LF}+\sigma_{LF}}{M_{LF}-\sigma_{LF}}$$

**Algorithm 1:** Defocus Blur Segmentation Map.
Let *Input* Image Defocus is represented by $D_{Img}$ and Output resultant segmented image is represented by IR${}_{seg}$
Transform $D_{Img}$ to $G_{Img}$$G_{Img(Rgn)}\leftarrow F\varkappa \left(G_{Img}\right)$*Max*${}_{F(\mathrm{Rgn})}\leftarrow M_{x}(G_{Img(Rgn)}$)*Min*${}_{F(\mathrm{Rgn})}\leftarrow Mn(G_{Img(Rgn)}$)*Mean*${}_{F(\mathrm{Rgn})}\leftarrow avgG_{Img(Rgn)}$)LTP sharpness formula applied for calculating sharpness estimation using Equation (14).Determine the PCNN initial parameters by adopting the initialization formula using Equation (20) to Equation (34).**for** the position of pixel *xy* in **$D_{Img}$ do****if**  $G_{Img_{\left(xy\right)}}>{Td}_{LTP}$ **then**$B_{LTP(xy)}\leftarrow 0$;**else**$B_{LTP(xy)}\leftarrow G_{Img}\left(\mathrm{xy}\right)$;**end if****end for**$M_{Edge}\leftarrow 0,f\leftarrow 0,P_{Con}\leftarrow 0\;\mathrm{and}\;n\leftarrow 1$**for** the position of pixel *xy* in $B_{LTP}$ **do**calculate $F_{xy}\left(n\right),L_{xy}\left(n\right),U_{xy}\left(n\right),E_{xy}\left(n\right),\Upsilon_{xy}\left(n\right)$**If**$\Upsilon_{xy}\left(n\right)==0$**then**$M_{Edge}\left(X,Y\right)\leftarrow 1,f\left(X,Y\right)\leftarrow 1$;**else**$M_{Edge}\left(X,Y\right)\leftarrow 0,f\left(X,Y\right)\leftarrow 0$;**end if****end for****for** the position of pixel *xy* in Υ **do****if**$\Upsilon_{xy}==1$**then****Lp** ← wblabel(Υ);**end if****end for****for** each *xy*: position of the pixel in $P_{Con}$ **do**$P_{Con\;xy}$ = the connectivity of the position *xy* in **Lp****end for**n ← n+1**for** the position of pixel *xy* in $M_{Edge}$ **do****if**$M_{Edge(xy)}>$***Td***$M_{Edge(xy)}$← 1;**else**$M_{Edge(xy)}\leftarrow 0$;**end if****end for**$\lambda \leftarrow B(M_{Edge})$**for** the position of pixel *xy* in **λ do****if**$\lambda_{xy}==1$$\lambda h_{xy}\leftarrow \lambda h_{xy}$;**else**$\lambda h_{xy}\leftarrow 0;$**end if****end for****return***Output*


We consider the two criteria for the $M_{HF}$ region; at first, the neuron firing at the ${nf}_{3}$th iteration, that refers to the pixel value of gray-scale $M_{HF}+\;\sigma_{HF}$. Secondly, the neuron firing at the ${nf}_{4}$th iteration, that refers to the pixel value of gray-scale $M_{HF}-\;\sigma_{HF}$. The parameters fulfill the following inequality criteria described in Equation (25) to Equation (27):

(25) $$M_{HF}+\sigma_{HF}\geq {ℑ}_{E}e^{-\left({nf}_{3}-1\right)F_{E}}$$

(26) $$M_{HF}-\sigma_{HF}\geq {ℑ}_{E}e^{-\left({nf}_{4}-1\right)F_{E}}$$

(27) $${nf}_{4}-{nf}_{3}>C$$

To solve Equation (25) to Equation (27), the result in Equation (28) is given below:

(28) $$C<\frac{1}{F_{E}}ln\frac{M_{HF}+\sigma_{HF}}{M_{HF}-\sigma_{HF}}$$

As per the classification criterion, the overview of Equations (25) and (28) is illustrated below in Equations (29) and (30):

(29) 0≤C≤8

(30) $$C=min\left\{8\left\lfloor \frac{1}{F_{E}}\left.\ln\frac{M_{HF}+\sigma_{HF}}{M_{HF}-\sigma_{HF}}\;\right\rfloor \right.\right\}$$

A specific reason for firing a particular neuron at the $M_{MF}$ the region is the 3×3 neighboring neurons firing and coupled in the early iteration. In the ${nf}_{5}th$ iteration, the gray-level $M_{MF}$ neurons are fired at once in the 3×3 neighbor. Such kind of neurons firing at ${nf}_{6}th$ iteration is the output of pulse-coupling. The parameters simplifications are specified by Equations (31)–(33):

(31) $$M_{MF}\geq {ℑ}_{E}e^{-\left({nf}_{5}-1\right)F_{E}}$$

(32) $$M_{MF}-\frac{2C}{3}\delta M_{MF}\geq {ℑ}_{E}e^{-\left({nf}_{6}-1\right)F_{E}}$$

(33) $${nf}_{6}-{nf}_{5}=1$$

Simplifying Equations (31)–(33), and the expression is given below in Equation (34):

(34) $$\delta =\frac{3\sigma_{LF}}{C(M_{LF}-\sigma_{LF})}$$

The focused parameters ${ℑ}_{E}$,${Td}_{min},F_{E},C\;\mathrm{and}\;\delta$ in the proposed algorithm are allocated adaptively corresponding to the pre-processing outcome of the LDoF images.

#### 3.1.2. Image Pre-Processing

In defocus image segmentation, a sample input image ($D_{Img}$) is applied that contains in-focused and unfocused regions. If ($D_{Img}$) is a color image, then in the first step, the gray-scale image ($G_{Img}$) will be produced from a color image. Maintaining the image’s local structure, for this purpose, the median filter function is used in the next step for overcoming the factor noise, and monotonic illumination transformation of in-focused and blurred smooth region. The median filter function is given below in Equation (35):

(35) $$G_{img(MF)}=medflt\left(G_{img(M)}\right)$$

#### 3.1.3. LTP Mask Based Sharpness Production

The Local Ternary Pattern (LTP) metric is used to calculate the sharpness mask for a patch of a local image observing each image pixel. The image in-focused region is depicted in white, as shown in Figure 2. The sharpness metric is calculated by applying the constant time of each pixel for a constant I and R. The LTP sharpness matrix is adopted for image analysis. Afterward, an estimated measure of the sharpness mask is used to produce a sharpness mask. An out-of-focused image is decomposed into tiny patches, and the estimated sharpness is performed per image patch. The process is completed, and therefore, the LTP values of each patch are estimates; subsequently, these values are used to classify each patch either as blur or non-blur. After performing the whole image looping, the image patches and blur and non-blur segments will be yielded using the sharpness metric.

#### 3.1.4. Out-of-Focused Estimation

Out-of-focused estimation is the scheme used for blurry content estimation in out-of-focused images. The blurred pixel values and non-blurred pixel values are characterized by applying the LTP algorithm. This pixel data is used to perform out-of-focused estimation; consequently, the pixels in blur classification are transformed by zero intensity values. The scheme is capable of the segmentation to proceed on distinct pixels.

**Figure 5:**
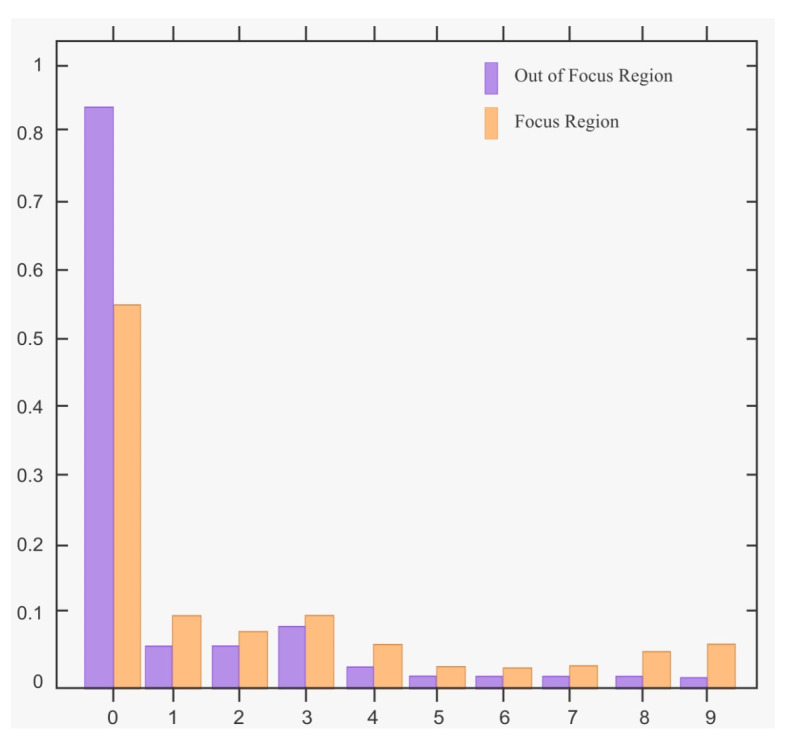
Histogram pattern in blurred and non-blurred regions.

A defocus blurred dataset [12] is online available for the public that contains 1000 semi blurred natural images following the ground-truth images, out of which 100 randomly selected images are used for finding the pattern distribution in blurred and non-blurred regions. The nine uniform histogram patterns of blurred and non-blurred regions are illustrated in Figure 5 in which the horizontal side consists of LTP histograms while the vertical axis indicates the pattern distribution. It is observed from the graph that the frequency distribution of 6 to 9 patterns in the non-blurred region is prominently high compared to the blurred one. Conversely, the 0 to 5 patterns abundant in the blurred region than the non-blurred region. It can be observed that the intensities of the central pixel $z_{c}$ and its neighboring pixels are the same in the flat image region, whereas these may vary in image in-focused area.

#### 3.1.5. PCNN Scheme

The PCNN structure produces prominent and accurate defocus-blur segmentation results in images even in noisy and blurry contents. It is noticed that in the existence of overlapped and contiguous regions, the PCNN can yield promising segmentation of defocused regions if only some specific conditions are fulfilled. It is observed that in highly prominent segmentation, every pixel is appropriately placed in a particular region where it belongs to. The generic scheme of image segmentation is applied to PCNN for its network parameters adjustment. Hence, neurons of specific region pixels are pulsed simultaneously, whereas the neurons of neighboring region pixels are not activated. The pulse-based neuron network used their connecting and feeding inputs. It is to be noted that the feeding input of a neuron is equivalent to its subsequent pixel intensity. Since the neurons have to capture phenomenon capability, the neurons linked to each spatially linked pixel and parallel intensities are managed to pulse altogether. The neighboring synchronous pulsing neuron set can categorize the defocus-blur image segmentation algorithm. The defocused segmentation using PCNN can be performed on different regions; for example, it can be the whole region, or a section of a deblur region, several regions, sub-regions, and their unions of the sample image. The preferable goal is the selection of network parameters so that each of the segments correctly relates to an entire region in the defocus blur image. The obtained segmentation quality depends on such kind of parameters. Contrarily, it is not always possible for PCNN to yield accurate and prominent segmentation using natural images. Therefore, it is required some advanced post-processing phases for splitting-merging segmentation.

**Figure 6:**
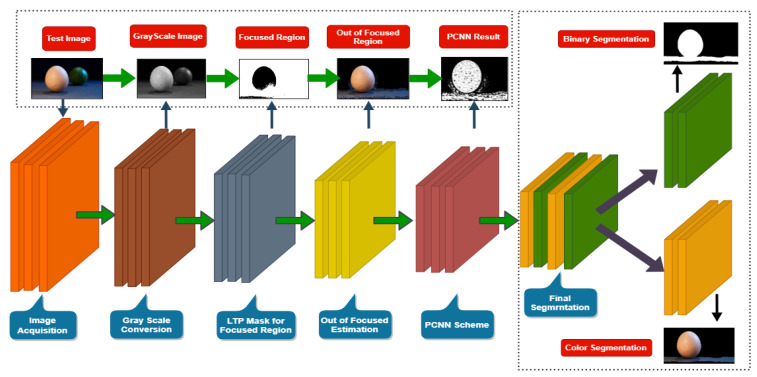
The Block diagram of the proposed scheme is illustrated. The primary points are displayed on the left side, whereas the right side of the figure represented the production and role of each image in the proposed algorithm.

In this scheme, at first, the sample image is transformed into a gray level. Next, the analysis is performed on this obtained image for the extraction of low-level frequency (${low}_{frq}$), middle-level frequency (${mid}_{frq}$), and high-level frequency (${high}_{frq}$) regions in the domain of frequency. These all frequency regions are measured to find out their mean value. These values are used as the preliminary parameters for PCNN execution on the transformed gray-level image. The PCNN module is implemented per pixel in the defocus-blur image; an edge-level segmented image is produced after finalizing the whole sequence of firing. This edge-based segmented image is merged and converted into a binary image to achieve the resultant out-of-focus image segmentation, as reported in Figure 6.

## 4. Defocus-Blur Segmentation Evaluation

We tested our proposed scheme using a public database [12], which contains 1000 natural blurry images of numerous classes, such as animals, beaches, humans, airships, trees, natural sceneries, and ships. It is observed that certain images in the database are challenging for performing in-focused segmentation schemes. The database is considered the top one amongst the other semi blur datasets, and each of the images contains the referenced segmented images. The database consists of various categories accompanying numerous human-made ground-truth de-blur images, also used to evaluate the proposed scheme. The database is categorized into six diverse sub-categories for further evaluation of in-focused segmentation.

### 4.1. Evaluation

**Figure 7:**
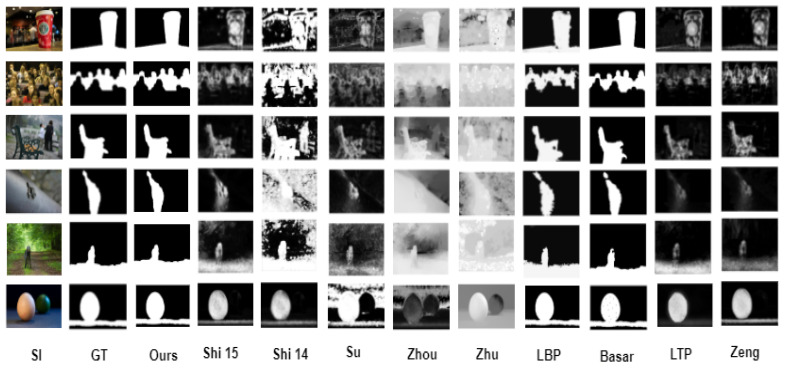
Defocus blur segmentation result presented by various defocus algorithms stated as Shi15 [14], Shi14 [16], Su [17], Zhou [18], Zhu [19], LBP [20], Basar21 [21], LTP [22], Zeng [23].

In this section, the scheme proposed is compared with other approaches in the study for validation. The images included in the database were segmented partially into blurred and non-blurred regions by applying the scheme described in Section 4. The scheme proposed for each comparator technique is presented in Figure 7 and the resultant images are in the range [0, 1]. The proposed scheme categorized the patches into blurred and non-blurred regions, as illustrated in Figure 7. The blur region is corresponded to the black color and is assigned a 0-pixel value, whereas the non-blur is denoted by white color and allocated the pixel value of 1. The in-focussed objects are prominent in the resultant out-of-focus image, while the unfocused one is not observable. The proposed scheme attributed the major errors, and those errors were identified as the substantial shortcomings of the Shi14 et al. [16], Zeng et al. [23], and Su et al. [17] (Section 2). The results achieved have a high resemblance to the GT images and noisy free backgrounds, whereas the referenced defocus approaches yield noisy backgrounds. In Shi14 et al. [16], Su et al. [17], and Zhu et al. [19], their blurred and non-blurred regions are mixed, which makes the objects invisible in the resultant images. Hence, the proposed scheme is highly robust for differentiating the blurry backgrounds compared to the previous studies.

The comparison is performed between the proposed scheme along with the 9 comparator techniques precisely described in Section 2. Yi et al. [20] estimated the sharpness measure $m_{LBP}$ following the ${Th}_{LBP}$. Su et al. [17] estimated the sharpness map by applying $m_{SVD}$. Zeng et al. [23] used the multiple conv-nets based technique of feature learning for defocus-blur detection. Shi14 et al. [16] adopted the $m_{GHS},\;m_{k},\;m_{LDA},\;m_{APS}$ jointly following a Naïve Bayes classifier and a multi-scale inference model. Shi15 et al. [14] shaped a sparse form representation about the image local patches adopted a pre-trained dictionary for a considerable identification of perceivable blur. Zhuo et al. [18] measured a depth map which is based on the edges’ width. Zhu et al. [19] evaluated the PSF-based model of the confined spectrum of frequency of the gradient area.

The results of these schemes are gray-level images where the highest level of intensity identifies the maximum sharpness, and other techniques apart from Zhu used a simple thresholding method, ${Td}_{seg}$ in the last step for yielding a segmented image, as indicated in the proposed algorithm. The parameters involved in the previous study were applied in their originally implemented code. In the meantime, obtaining the actual code for Zhu’s technique was unfeasible, and the main reason is its involvement in Adobe System Inc. The proposed algorithm generated the outputs reported in this research. The depth map was standardized by the range [0,8] and obtained the sharpness map by inversion. In the related study, most of the authors adopted the performance measures, i.e., precision, recall, and F1-score, for evaluating the defocus blur segmentation algorithms. The proposed algorithm following the comparator approaches also used these measures, moreover, few more metrics, such that accuracy metric, Matthew’s Correlation-Coefficient (MCC), Jaccard-Coefficient Measure (JCM), Dice-Similarity-Coefficient (DSC), and Specificity are all applied in this research and not applied in mostly alternate algorithms. The performance measures are described as given below:

#### 4.1.1. Precision and Recall

Precision and recall yields for defocusing the segmented algorithm to vary the ${Td}_{seg}$ sampled at each integer value applying the interval [0, 255] for producing the final de-blur segmentation of the estimated sharpness depicted in Equation (36) and Equation (37).

(36) $$\;\mathrm{Precision}\;=R_{sg}\cap R_{gt}/R_{sg}$$

(37) $$\;\mathrm{Recall}\;=R_{sg}\cap R_{gt}/R_{gt}$$

where $R_{sg}$ represents the pixels set in the blurry region of segmentation, $R_{gt}$ symbolizes the pixels set in the blurred region of ground truth. Our proposed scheme generated a notable precision, i.e., 0.9894 other than the previous works, while the recall value is 1. Furthermore, the presented algorithm produces the prominent output than the comparator’s techniques.

#### 4.1.2. Accuracy

The segmentation accuracy is measured using the confusion matrix and measured by disseminating the whole number that accurately classifies pixels by the total number of reference pixels. Likewise, the accuracy of certain classifications is computed by a certain quantity of accurately categorized pixels in every column of the matrix. The accuracy identifies the learning set of pixels of specified types of cover, categorized and determined by distributing the number of accurately classified pixels of the training dataset number and also applied in numerous classes. The accuracy represents the orderly errors, whereas the precision corresponds to unsystematic errors. True Positive (*TP*) leads its segmentation result to 1 while the ground-truth output also indicates 1; True Negative (*TN*) denotes the segmentation result, as well as the ground-truth output; both are equal to 0. False Positive (*FP*) represents one which is the result of segmentation, whereas 0 is the result of *GT*. Contrarily, False Negative (*FN*) identifies its segmented output tends to 1, while the *GT* also represents 1, and these expressions are used for calculating the accuracy, and also calculates the total number (*N*) of covers. The segmented accuracy of the presented result is 0.9991 as already illustrated in Figure 10. The obtained ratio of the result is described in Equation (38) as given below:

(38) $$\mathrm{Accuracy}\;=\frac{NTP+NTN}{TP+FN+FP+TN}$$

#### 4.1.3. F1 Measure

**Figure 8:**
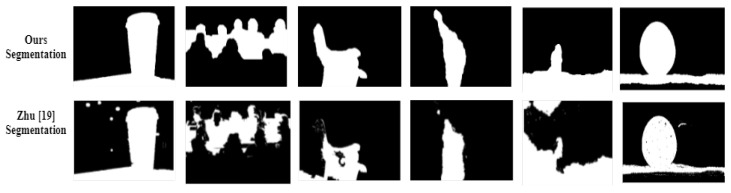
Binary defocus segmented comparison along with Zhu et al. [19].

**Figure 9:**
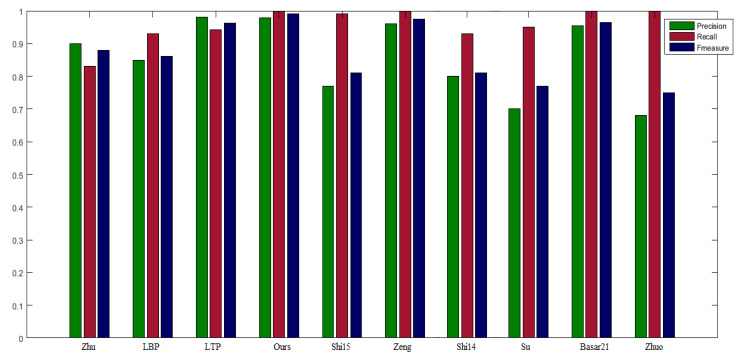
Results comparison of Precision, Recall, and F1-score for using adaptive thresholds. Zhu [19] applied the graph cut method instead of simplified thresholding. LTP [22] algorithm is obtained by applying a lower threshold value, i.e., ${Td}_{seg}=0.35$. Our proposed scheme can obtain comparative result (precision = 0.9894, recall = 1, F1-measure = 0.9990). The other comparative methods used are stated as LBP [20], Shi15 [14], Zeng [23], Shi14 [16], Su [17], Basar21 [21], Zhuo [18].

The threshold-based adaptive scheme presented by [58] for defocus blur segmentation following the threshold described in Equation (39) as given below:

(39) $${Td}_{seg}=\frac{2}{X\times Y}\sum_{m=1}^{X}\sum_{n=1}^{Y}I_{map}\left(m,n\right)$$

where, *X, Y* are signifying as the width as well as the height of the sharpness estimation $I_{map}$. Therefore, in [59], it is stated that F1-score is using for evaluating the test accuracy. The expressions of precision and recall were estimated by using F-score for comparison purposes as mentioned in Equation (40) as given below:

(40) $$F_{\kappa }=\frac{\left(1+\kappa^{2}\right)\times \;\mathrm{precision}\;\times \;\mathrm{recall}}{(\kappa^{2})\times \;\mathrm{precision}+\;\mathrm{recall}}$$

where, $\kappa^{2}$ was allocated the value 0.3 which is stated in [59]. Zhu et al. [19] generated the segmented result by using the graph cut method instead of applying thresholding depth-map. The same parameters are used in the proposed scheme as recommended in that mentioned paper, such that ${℧}_{0}=1000,\;{℘}_{℧}=0.04,\;t=2.$ Conversely, the parameters in the PCNN structure is allocated as: $W_{con}=\left[0.5,\;1,\;0.5;1,\;0,\;1;0.5,\;1,\;0.5\right],\;\kappa =5,$ slide window = 110 by 110, neighborhood pixels = 3 by 3. Exemplar segmented images maps in Figure 7 are depicted in Figure 8. Figure 9 is all about the comparison graph that describes the visible difference of the proposed scheme with reference to comparator studies. The bar graph visibly displays that the proposed scheme outperforms than existing techniques: precision =0.9894, recall =1, F1-score =0.9990.

#### 4.1.4. Matthew’s Correlation-Coefficient (MCC)

**Figure 10:**
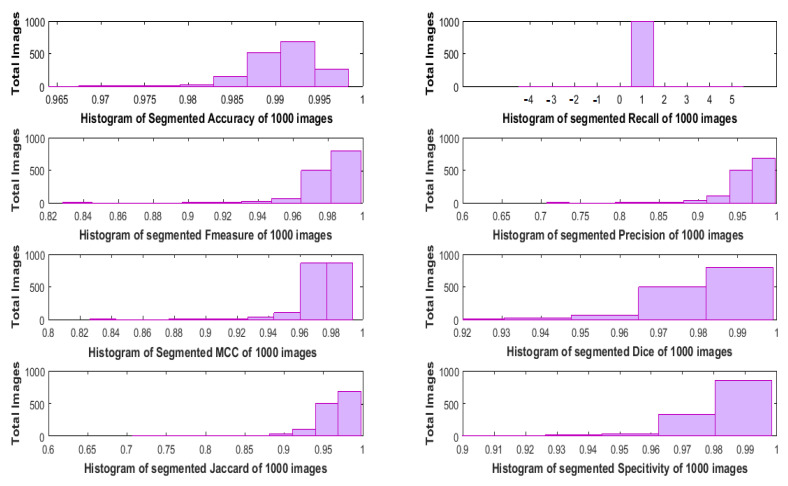
Numerous performance metrics are represented in histogram structures using overall 1000 partially-blurred images of the defocused-blur dataset.

MCC is one of the measures for evaluating the similarity ratio of two binary images, i.e., the output and ground-trith image. Its output lies under the interval [−1, 1], whereas −1 identifies the incorrect output and 1 denotes the correct result. It is observed that MCC provides more descriptions compared to F-measure and is also more accurate for binarized segmentation as it considers the overall proportion of the confusion matrix, i.e., *TP, FP, TN, FN*. The suggested MCC value is 0.9870 as depicted in Figure 10 and also reported in below Equation (41):

(41) $$MCC=\frac{\left(TP\right)\times \left(TN\right)-\left(FP\right)\times \left(FN\right)}{\sqrt{\left[TP+FP\right]\left[TP+FN\right]\left[TN+FP\right]\left[TN+FN\right]}}$$

where *TP* identifies True Positive, *TN* represents True-Negative, *FP* and *FN* denote False Positive value and False Negative one, respectively.

#### 4.1.5. Jaccard-Coefficient Measure (JCM)

JCM is the opposite of Jaccard-Distance (JD) that measures the similarity ratio between the segmented as well as the GT images. Both of the vectors $u=(u_{1}+u_{2},\cdots u_{n})$ and $v=(v_{1}+v_{2},\cdots v_{n})$ are real, i.e., $\left(u_{i},v_{i}\right)\geq 0$ as described in Equation (42) as follows:

(42) $$J\left(u,v\right)=\frac{\sum_{j}\min(u_{j},\;v_{j})}{\sum_{j}\max(u_{j},\;v_{j})}$$

However,$\;J\left(U,V\right)=1.0\leq J\left(U,V\right)\leq 1$ if and only if *U* and *V* both are empty. The *JCM* values included under the interval [0, 1], already reported in Figure 10. The value 0 represents lower similarity, whereas 1 denotes the higher similarity between the two binary images.

#### 4.1.6. Dice-Similarity-Coefficient (DSC)

As the name indicates, *DSC* is applied for calculating the similarities of the labeled regions of about two binarized images. DSC calculation is applied frequently in the whole process of segmentation to calculate the performance of DSC along with the effective weighting of the instance. The instance values are involved in the range of 1 and 0. If the DSC output is 1, it indicates the accuracy, while 0 represents the inaccurate output. The presented DSC output is 0.9891 as illustrated in Figure 10. It describes in Equation (43) as below:

(43) $$DSC=\frac{2TP}{2TP+FP+FN}$$

#### 4.1.7. Specificity

The expected test output analyzes in particular outputs without depicting the False-Positive output identifies the specificity’s numerical value. The estimated specificity result is 0.9988. It is explainable in the below Equation (44):

(44) $$\mathrm{Specificity}=\frac{TN}{TN+FP}$$

The running time comparator of the proposed scheme corresponding to the alternate segmentation approaches is reported in Table 3. The running time of our overall proposed scheme is calculated on the proposed hybrid algorithm (PCNN and LTP) and ranking method.

The histogram-based numerous evaluating metrics of the defocus-blur dataset contains 1000 blurry images are displayed in Figure 10. Among the histogram bins of the entire dataset, the resultant recall value is 1, which tends to be an accurate defocus-blur segmentation. Additionally, the output values of other metrics, i.e., F1-score, Dice, and Specificity, are one or around one. Moreover, the resultant accuracy value of most images in the dataset is approximately 0.99. Furthermore, the output histogram’s values of mostly images about Precision and Jaccard are lying under 1. Similarly, the MCC’s histogram result about a defocus-blur dataset based on 1000 images is almost 0.98 value. It is summarized that the output values of most of the metrics are approximately 1. Henceforth, it is proved that the proposed scheme produces accurate defocus-blur segmentation compared to the referenced approaches.

**Figure 11:**
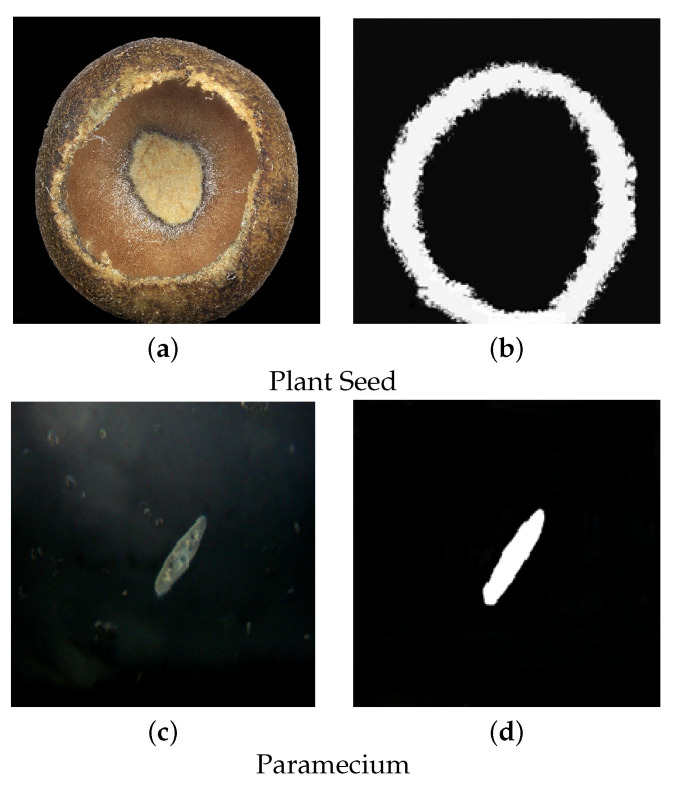
The proposed scheme is applied to microscopic images. (**a**) is about original input image of a Plant Seed while its segmentation map is illustrated in (**b**). (**c**) is the original input image of Paramecium and (**d**) is the segmented Paramecium image.

Figure 11 shows examples of the microscopic images other than our defocus-blur dataset. The first image is about a plant seed [76] which has a roughly round shape. Its result is in a ring-like shape of the sharp region, while the rest are illustrated as blur regions. On the other hand, another image is underwater paramecium in the sharp region, while the microorganisms in the background are depicted as blur regions. The blurred objects are hidden in black in the output image, and the sharp one is visible in the white region.

Table 4 represents the performance estimation of our study following the referenced techniques. According to Table 4, Basar21 et al. [21] and the proposed scheme has the highest performance estimation compared to existing approaches. LTP [22] and Zeng et al. [23] achieved high rank, Precision, and F-measure, whereas Zhuo and Sim [18], and Zeng et al. [23] reported the highest Recall than referenced methods. The high-rank precision is recorded by Zhu et al. [19], while lower in Recall and F-measure. LBP [20], and Shi14 et al. [16] illustrated the high rank Recall, whereas lower in terms of Precision and F-measure values. The low rank Precision, and F-measure is recorded by Su et al. [17], and Shi15 et al. [14], while achieving the high Recall estimation. Zhuo and Sim [23] obtained the lowest Precision and F-measure compared to others.

### 4.2. Ranking Evaluation

In this research, the Evaluation Based on Distance from Average Solution (EDAS) scheme [21,69,74] is applied to evaluate the proposed scheme ranking following the numerous comparators concerning efficiency and accuracy. The proposed scheme is compared with existing techniques based on different performance estimations here. In our study, the EDAS approach is represented by the authors for accumulating the cross-efficient results of different parameters of the overall ten methods, containing ours as well. The EDAS ranking is applied here consists of 3 performance-estimations only, such as Precision, Recall, and F1-score, whereas the rest are used by the proposed scheme only. The cumulative value of Appraisal-Scores $\left({ℜ}_{a}\right)$ is estimated for ranking of alternate approaches to estimate the positive value of the distance from the mean solution is symbolized as ($P_{\vartheta }$) and negative value of the distance from the mean solution is symbolized as ($N_{\vartheta }$).

In Table 5, given below, the performance estimations are observed as the criteria of previous approaches.

**Step 1**: Determine the average value (£) solution of overall metrices in expresion (45);

(45) $$\left(£\right)={\left[{£}_{b}\right]}_{1\times \partial }$$

where,

(46) $$\left({£}_{b}\right)=\frac{\sum_{i=1}^{n}\chi_{ab}}{n}$$

The step mentioned above calculates the performance estimations as different algorithms criteria. The cumulative measure of Equations (45) and (46) can be achieved as the mean value $\left({£}_{b}\right)$ for each value of criteria measured in Table 5.

**Step 2**: This step determines the positive distance values from mean value $\left(P_{\vartheta }\right)$ in Equations (47)–(49) as given as follows:

(47) $$P_{\vartheta }={\left[{\left(P_{\vartheta }\right)}_{ab}\;\right]}_{\partial \times \partial }$$

If the *b*th criterion is more valuable then

(48) $${\left(P_{\vartheta }\right)}_{ab}=\frac{\mathrm{Maximum}\;(0,\;\;\;\left(\psi_{\vartheta_{b}}-\chi_{ab}\right))}{\psi_{\vartheta_{b}}}$$

and if non-valuable then the equation will be changed as given below:

(49) $${\left(P_{\vartheta }\right)}_{ab}=\frac{\mathrm{Maximum}\;(0,\;\;\;\left(\chi_{ab}-\psi_{\vartheta_{b}}\right))}{\psi_{\vartheta_{b}}}$$

The outputs revealed in Table 6 are given as follows:

**Step 3**: The negative values of distances are calculated in this step from average $(N_{\vartheta }$) using expressions (50), (51), and (52) as given below:

(50) $$\left(N_{\vartheta }\right)={\left[{\left(N_{\vartheta }\right)}_{ab}\right]}_{\partial \times \partial }$$

If the *b*${}_{th}$ criteria is more valuable compared to the following expression (51) is determined:

(51) $${\left(N_{\vartheta }\right)}_{ab}=\frac{\mathrm{Maximum}\;(0,\;\left(\psi_{\vartheta_{b}}-\chi_{ab}\right))}{\psi_{\vartheta_{b}}}$$

and if non-valuable then the mentioned expression will be modified in expression (52) as follows:

(52) $${\left(N_{\vartheta }\right)}_{ab}=\frac{\mathrm{Maximum}\;(0,\;\;\;\left(\chi_{ab}-\psi_{\vartheta_{b}}\right))}{\psi_{\vartheta_{b}}}$$

whereas the ${\left(P_{\vartheta }\right)}_{ab}$ and ${\left(N_{\vartheta }\right)}_{ab}$ identified the positive distance value and negative distance value of *b*${}_{th}$ rated approaches from the mean value concerning *a*${}_{th}$ rating performance estimations, respectively.

The outputs revealed in Table 7 are as follows:

**Step 4**: Determine the cumulative sum of $\left(P_{\vartheta }\right)$ for the rated approaches in Table 8 as follows:

(53) $${\left({\mathrm{SP}}_{\vartheta }\right)}_{a}=\sum_{b=1}^{n}y_{b}{\left(P_{\vartheta }\right)}_{ab}$$

**Step 5**: Determine the cumulative sum of ${\left(N_{\vartheta }\right)}_{ab}$ for the rated approaches in Table 9 mentioned in Equation (54) as given below:

(54) $${\left({\mathrm{SN}}_{\vartheta }\right)}_{a}=\sum_{b=1}^{n}y_{b}{\left(N_{\vartheta }\right)}_{ab}$$

The output is revealed in the table below:

**Step 6**: This step normalizes and estimates the values of ${\left({\mathrm{SP}}_{\vartheta }\right)}_{a}$ and ${\left({\mathrm{SN}}_{\vartheta }\right)}_{a}$ for the rated approaches as mentioned in expressions (55) and (56):

(55) $$\Gamma {\left({\mathrm{SP}}_{\vartheta }\right)}_{a}=\frac{{\left({\mathrm{SP}}_{\vartheta }\right)}_{a}}{{\mathrm{Maximum}}_{a}\left({\left({\mathrm{SP}}_{\vartheta }\right)}_{a}\right)}$$

(56) $$\Gamma {\left({\mathrm{SN}}_{\vartheta }\right)}_{a}=1-\frac{{\left({\mathrm{SN}}_{\vartheta }\right)}_{a}\;}{{\mathrm{Maximum}}_{a}\left({\left({\mathrm{SN}}_{\vartheta }\right)}_{a}\right)}$$

**Step 7**: This step estimates the values of $\Gamma {\left({\mathrm{SP}}_{\vartheta }\right)}_{a}$ and $\Gamma {\left({\mathrm{SN}}_{\vartheta }\right)}_{a})$ to obtain an appraisal-score (*AS*) which is equal to $\left({ℜ}_{a}\right)$ for the ranked approaches specified as follows:

(57) $${ℜ}_{a}=\frac{1}{2}\left(\Gamma {\left({\mathrm{SP}}_{\vartheta }\right)}_{a}-\Gamma {\left({\mathrm{SN}}_{\vartheta }\right)}_{a}\right)$$

where $0\leq {ℜ}_{a}\leq 1$.

The $\left({ℜ}_{a}\right)$ is determined by the aggregate score of $\Gamma {\mathrm{SP}}_{\vartheta }$ and $\Gamma {\mathrm{SN}}_{\vartheta }$.

**Step 8**: Measure the appraisal-scores $\left({ℜ}_{a}\right)$ in terms of decreasing order, and then determine the ranking of appraised approaches. The highest$\left({ℜ}_{a}\right)$ indicates the best ranking method. Therefore, in Table 10, the proposed scheme has the largest$\left({ℜ}_{a}\right)$.

The final ranked result is represented in the table below:

**Table 10:** Analysis results of 10 state-of-the-art methods.

Approaches	${\left({\mathrm{SP}}_{\vartheta }\right)}_{a}$	${\left({\mathrm{SN}}_{\vartheta }\right)}_{a}$	$\Gamma {\left({\mathrm{SP}}_{\vartheta }\right)}_{a}$	$\Gamma {\left({\mathrm{SN}}_{\vartheta }\right)}_{a}$	${ℜ}_{a}$	Ranking
Zhu [19]	0.0329	0.0586	0.2114	0.4058	0.3086	**6**
Shi15 [14]	0.1150	0.0079	0.7389	0.9192	0.8291	**2**
Shi14 [16]	0.0703	0	0.4520	1	0.7260	**4**
Su [17]	0.0934	0	0.6002	1	0.8001	**3**
Zhuo [18]	0.1557	0.0083	1	0.9149	0.9574	**1**
Zeng [23]	0	0.0968	0	0.0179	0.0089	**9**
LBP [20]	0.0111	0	0.0714	1	0.5357	**5**
LTP [22]	0.0039	0.0940	0.0251	0.0466	0.0358	**8**
Basar21 [21]	0	0.0897	0	0.0905	0.0452	**7**
**Ours**	0	0.0986	0	0	0	**10**

Table 10 is the overview of EDAS ranking outputs that performs the comparison of alternate techniques based on the above three performance estimations. The ranking in Table 10 illustrates the proposed scheme is outperforming the comparator methods. According to the analysis reported in Table 10, our scheme is recorded at the top-ranked, whereas Zeng [23], and LTP [22] are included in the second and third rank, respectively. The methods on fourth and fifth ranks are occupied by Basar21 [21], and Zhu [19] whereas LBP [20], and Shi14 [16] are positioned on the sixth and seventh rank, respectively. Su [17], Shi15 [14], and Zhuo [18] are laid down in the eighth, ninth, and tenth rank, respectively.

### 4.3. Discussion

The overall performance of our algorithm may affect the presence of noise in images. Such problems can be overcome by adopting the filters for noise reduction and then applying the proposed scheme. The presented metric was expanded by statistical differentiation of PCNN and LTP using a collection of defocus images. Meanwhile, the resources of blurred regions are primarily the cause of defocus blur. Our suggested metric presently is only adept at detecting defocus blur. The blurriness is that caused by defects lenses and tools in imaging systems and motion blur, and it would be worth exploring the blurriness model mainly due to the optical system properties and also studying the properties of the different patterns such as LBP and LTP [62] on numerous types of blur regions. The images having smooth regions may also degrade the performance of the proposed algorithm. The EDAS based ranking approach is applied to improve the evaluation of the proposed scheme by using three primary metrics, Precision, Recall, and F1-score, which proved the proposed scheme on top of the rank.

## 5. Conclusions

The proposed hybrid scheme combines PCNN and LTP algorithms. After the focused region extraction, the firing sequence of the neurons consists of some required information of the defocused image features such as edge, texture, and region information. The presented algorithm illustrates the LTP pattern patches on the in-focused and blurred regions in the de-blurred image. The proposed scheme comprises the sequential firing nature of the PCNN neuron model following the criterion and design for classification of a pixel to establish critical parameters, along with the local sharpness map-based LTP algorithm. The proposed scheme detects the sharpest region of a defocused image and achieves high accuracy, and less execution than the reference algorithms explained in the related work section. Our proposed sharpness map estimates the distinct LTP patterns numerically in the localized neighborhood pixels. The overall EDAS ranking results reported that the suggested scheme is on top of the rank for further analysis and explanation. Henceforward, the whole experimental outputs and the evaluation result visibly illustrate the promising performance of the proposed approach compared to alternative approaches in terms of efficiency and accuracy in the area of defocus-blur segmentation. The future aim of the proposed study is to expand its scope in agriculture, medical object classification, and 3D defocus image estimation, and also GPU implementation would be preferred in the case of a large dataset instead of the CPU.

## Figures and Tables

**Table 1:** Fuzzy logic’s contributions in related research works.

Authors	Study Title	Algorithm Description
Basar20 et al. [69]	“Unsupervised color image segmentation: A case of RGB histogram-based K-means clustering initialization”	Proposed an initialization approach based on K-means to solve the image segmentation problem by applying the EDAS techniques for ranking purposes.
Ilieva et al. [70]	“Decision analysis with classic and fuzzy EDAS Modifications”	Suggested the L1 measure in EDAS method for solving some issues in MCDM problems to overcome the computational complexity.
Liang et al. [71]	“An Integrated EDAS-ELECTRE Method with Picture Fuzzy Information for Cleaner Production Evaluation in Gold Mines”	proposed a technique of 4-level degrees of membership with PFN (Picture Fuzzy numbers) to rank the construction of cleanser for gold mines.
Li et al. [72]	“Linguistic-Neutrosophic Multi-criteria Group Decision-Making Approach with EDAS technique”	The presented approach adopted the MCGDM (Multi-Criteria Group-Decision Making) scheme, which is based on EDAS for setting and controlling the neutrosophic issues.
Stevic et al. [73]	“Evaluation of Suppliers Under Uncertainty: A Multiphase Approach Based on Fuzzy AHP and Fuzzy EDAS”	The Fuzzy Analytical Hierarchy approach is implemented to indicate and estimate the suppliers and intended to explore the Fuzzy-EDAS model.
Mehmood et al. [74]	“A Trust-Based Energy Efficient and Reliable Communication Scheme (Trust-Based ERCS) for Remote Patient Monitoring in Wireless Body Area Networks”	The suggested study is about a stable technique for communication purposes to preserve the privacy of WBAN (Wireless Body Area Network). The evaluation of the method is performed by the EDAS approach and identified by the top-most rank.
Basar21 et al. [21]	“A Novel Defocused Image Segmentation Method based on PCNN and LBP”	The proposed hybrid presented a novel sharpness descriptor for segmenting the in-focused region from unfocused one in the defocused blur image and ranked on the top by adopting the EDAS ranking scheme.

**Table 2:** Symbols and descriptions used in our algorithm.

Symbol	Description
$D_{Img}$	Test image
PCNN	Pulse Coupled Neural Network
LTP	Local Ternary Pattern
$G_{Img}$	Gray-scale image
$IR_{seg}$	Resultant image
LDoF	Low Depth of Filed
MCC	Matthew’s Correlation-Coefficient
DSC	Dice-Similarity-Coefficient
JCM	Jaccard-Coefficient Measure
$G_{Img(Rgn)}$	Gray-scale image region
Max${}_{F(Rgn)}$	Maximum frequency region
Min${}_{F(Rgn)}$	Minimum frequency region
Mean${}_{F(Rgn)}$	Average frequency region
$D_{Img(xy)}$	The pixel coordinate (*x*,*y*) in the defocused image
${Td}_{LTP}$	Thresholded LTP value
$B_{LTP}$	Binary segmentation of LTP algorithm
$M_{Edge}$	Resultant edge map of PCNN
*f*	The sequence of firing matrix that records each firing order of pixel
$P_{Con}$	Pixel-connectivity matrix
$B_{seg}$	Binary segmented output
$C_{seg\;}$	Color segmented output
$W_{con}$	Connecting-weight matrix
δ	Connecting coefficient of strength
${ℑ}_{E}$	Dynamic-threshold coefficient
ϵ	Dynamic-strength coefficient
$F_{E}$	Decay-factor
$M_{LF}$	Gray-scale mean values of low scale frequency
$M_{MF}$	Gray-scale mean values of mid scale frequency
$M_{HF}$	Gray-scale mean values of high scale frequency
Td${}_{min}$	Threshold value along with minimum limit
C	Criteria for judgment
γ	Dynamic-threshold matrix
EDAS	Evaluation Based on Distance from Average Solution

**Table 3:** Comparative running time evaluation of numerous approaches. The time of the proposed scheme is based on PCNN and LTP algorithms.

Out-of-Focus Segmentation	Approx. Runtime
LBP [20]	27.19 s
LTP [22]	26.50 s
Shi15 [14]	38.36 s
Zeng [23]	19.18 s
Shi14 [16]	705.2 s
Su [17]	37.00 s
Zhuo [18]	20.59 s
Zhu [19]	12.00 min
Basar21 [21]	29.05 s
**Ours**	28.99 s

**Table 4:** Performance-Estimation of numerous approaches.

Approaches	Performance-Estimations		
	Precision	Recall	F1-Score
Zhu [19]	0.9632	0.8651	0.8681
Shi15 [14]	0.7664	0.9985	0.8653
Shi14 [16]	0.8001	0.9531	0.8112
Su [17]	0.7645	0.9644	0.7997
Zhuo [18]	0.6788	1	0.7667
Zeng [23]	0.9886	1	0.9895
LBP [20]	0.8631	0.9651	0.8697
LTP [22]	0.9981	0.9564	0.9811
Basar21 [21]	0.9785	1	0.9885
**Ours**	0.9894	1	0.9990

**Table 5:** Cross efficient Calculated values.

Approaches	Performance-Estimations		
	Precision	Recall	F1-Score
Zhu [19]	0.9633	0.8651	0.8681
Shi15 [14]	0.7664	0.9985	0.8653
Shi14 [16]	0.8001	0.9531	0.8112
Su [17]	0.7645	0.9644	0.7997
Zhuo [18]	0.6788	1	0.7667
Zeng [23]	0.9886	1	0.9895
LBP [20]	0.8631	0.9651	0.8697
LTP [22]	0.9981	0.9564	0.9811
Basar21 [21]	0.9785	1	0.9885
**Ours**	0.9894	1	0.999
${£}_{b}$	0.87907	0.97026	0.89388

**Table 6:** Evaluation results of average value $\left(P_{\vartheta }\right)$.

Approaches	Performance-Estimations		
	Precision	Recall	F1-Score
Zhu [19]	0	0.1083	0.02884
Shi15 [14]	0.1281	0	0.0319
Shi14 [16]	0.0898	0.0176	0.0924
Su [17]	0.1303	0.0060	0.1053
Zhuo [18]	0.2278	0	0.1422
Zeng [23]	0	0	0
LBP [20]	0.0181	0.0053	0.0270
LTP [22]	0	0.0142	0.0270
Basar21 [21]	0	0	0
**Ours**	0	0	0

**Table 7:** Evaluation results of average $(N_{\vartheta }$).

Approaches	Performance-Estimations		
	Precision	Recall	F1-Score
Zhu [19]	0.0957	0	0
Shi15 [14]	0	0.0291	0
Shi14 [16]	0	0	0
Su [17]	0	0	0
Zhuo [18]	0	0.0306	0
Zeng [23]	0.1245	0.0306	0.1069
LBP [20]	0	0	0
LTP [22]	0.1354	0	0.0975
Basar21 [21]	0.1131	0.0306	0.1058
**Ours**	0.1255	0.0306	0.1175

**Table 8:** Analysis outputs of the calculated aggregate $\left(P_{\vartheta }\right)$.

Criteria (W)	0.6125	0.2737	0.1139	${\left({\mathrm{SP}}_{\vartheta }\right)}_{a}$
Approaches	Performance-Estimation			
	Precision	Recall	F1-Score	
Zhu [19]	0	0.0296	0.0033	0.0329
Shi15 [14]	0.0785	0	0.0036	0.1150
Shi14 [16]	0.0550	0.0049	0.0105	0.0703
Su [17]	0.0798	0.0016	0.0121	0.0934
Zhuo [18]	0.1395	0	0.0162	0.1557
Zeng [23]	0	0	0	0
LBP [20]	0.0111	0	0	0.0111
LTP [22]	0	0.0039	0	0.0039
Basar21 [21]	0	0	0	0
**Ours**	0	0	0	0

**Table 9:** Analysis outputs of the calculated aggregate $\left(N_{\vartheta }\right)$.

Criteria (W)	0.6125	0.2737	0.1139	${\left({\mathrm{SN}}_{\vartheta }\right)}_{a}$
Approaches	Performance-Estimation			
	Precision	Recall	F1-Score	
Zhu [19]	0.0586	0	0	0.0586
Shi15 [14]	0	0.0079	0	0.0079
Shi14 [16]	0	0	0	0
Su [17]	0	0	0	0
Zhuo [18]	0	0.0083	0	0.0083
Zeng [23]	0.0763	0.0083	0.0121	0.0968
LBP [20]	0	0	0	0
LTP [22]	0.0829	0	0.0112	0.0940
Basar21 [21]	0.0692	0.0083	0.0121	0.0897
**Ours**	0.0768	0.0084	0.0134	0.0986

## Data Availability

Not applicable.
